# Numerical Simulation of Motion and Distribution Characteristics for Electrospray Droplets

**DOI:** 10.3390/mi14020396

**Published:** 2023-02-05

**Authors:** Jiaxin Jiang, Zunxu Qian, Xiang Wang, Huatan Chen, Guoyi Kang, Yifang Liu, Gaofeng Zheng, Wenwang Li

**Affiliations:** 1 School of Mechanical and Automotive Engineering, Xiamen University of Technology, Xiamen 361024, China; 2 Department of Instrumental and Electrical Engineering, Xiamen University, Xiamen 361102, China; 3 Shenzhen Research Institute of Xiamen University, Shenzhen 518000, China

**Keywords:** electrospray, charged droplets, numerical simulation

## Abstract

Electrospray is a typical technology to prepare large amounts of droplets at micro/nano scale. Establishing the relationship between the processing parameters and the motion and distribution characteristics for electrospray droplets is an effective approach to guide the uniform deposition of the electrospray membrane. In this paper, a dynamic model of electrospray droplets based on the fully resolved direct numerical simulation (FR-DNS) method was constructed, and the spatial motion behaviors of charged droplets were simulated. The coupling effect of electric field force, the charge repulsive force, and the gravity on the motion and distribution of electrospray droplets was studied, and the relationship between processing parameters including the applied voltage and distance from the nozzle to the collecting plate and the spatial distribution of charged droplets was clarified in a direct way. The simulation model provided a good approach for the quantitative description of the motion and distribution behaviors for electrospray droplets, which would help to guide the control of the electrospray jet ejection process.

## 1. Introduction

Liquid spray [1,2] is a phenomenon occurring via the cohesive properties of liquids, which usually happens in industrial fields including rocket combustors, internal combustion engines, nuclear fission, and agricultural irrigation. Electrospray [3,4] is an emerging technology in the generation of nano/micro particles with smaller diameters. With the coupling effect of high electrical field force, a liquid solution can be atomized into large amounts of droplets ranging from hundreds of micrometers down to several tens of nanometers with nearly monodisperse distribution, which has been widely used in the fields of flexible electronics [5,6], biomedicines [7,8], battery separators [9,10], et al.

Electrospray is a process with multi-physics coupling, and the interaction effect among a large number of droplets is complex. The high charge density on the surface of electrospray droplets leads to a strong charge repulsive force among the charged droplets, resulting in the irregular motion and distribution of electrospray droplets [11,12]. The physical properties of the solution including the dielectric constant, the viscosity, and the surface tension play an important role in the formation of electrospray droplets, which have been extensively studied in the literature. Fukuda [13] connected the collecting plate to a Coulomb meter to measure the electric charge. The physical properties of the electrospray solution were considered on the droplet charge, finding that the charge per unit volume was distributed from 10^−3^~1.3 C/kg. Rubio [14] established a three-plate experimental system with high-speed video cameras to measure the droplet diameter and the electric charge, of which the droplets were mounted on an optical table with a pneumatic anti-vibration isolation system. The experimental results indicated that the electric charge on a single droplet was in the femtocoulomb scale, and the diameter of the electrospray droplet was several micrometers. Identifying the distribution characteristics and interaction mechanisms of electrospray droplets is important for the uniform and controllable deposition of electrospray membranes. To accurately describe the electrospray process, several works have been reported about the numerical simulation model. Herrada [15] assumed that all the free electrical charges were distributed over the liquid–gas interface to describe the flow pattern within the entire liquid domain, which was verified by the experimental results. Dong et al. [16] investigated the effects of electric field distribution and intensity on the electrospray process and resultant microsphere diameter, indicating that from higher electric field strength comes smaller microsphere diameter due to the fission caused by the surface charge. Dastourani et al. [17] conducted a two-phase numerical simulation to investigate the temporal and spatial evolutions of the cone-jet mode in an electrospray process in connection with the operating parameters, revealing the mechanism of operating parameters on the altering of the flow configuration. Ouedraogo [18] proposed a numerical approach with a multiphase flow equation coupled with an electro-quasistatic problem including the capacitive, the resistive, and the convective electric currents, and sufficient spatial resolution for electrospray droplets was achieved. However, the direct relationship between processing parameters and droplet motion and distribution behavior is still required to be further investigated by the simulation method to guide the control of the electrospray jet ejection process.

In this paper, a dynamics model of electrospray droplets was constructed to identify the mechanism of processing parameters on the spatial motion behaviors of charged droplets. The effects of applied voltage and distance from the nozzle to the collecting plate on the distribution of electrospray droplets were investigated.

## 2. Dynamic Model of Electrospray Droplets

### 2.1. Force Analysis on Electrospray Droplets

**Figure 1:**
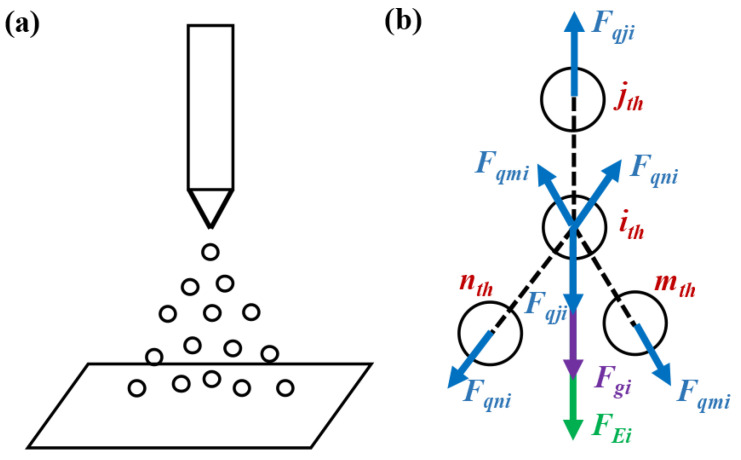
Force analysis on electrospray droplets: (**a**) schematic diagram of spatial distribution of electrospray droplets; (**b**) force analysis on the *i_th_* droplet. *F_Ei_*: the electric field force on the *i_th_* droplet; *F_qji_*, *F_qmi_*, and *F_qni_*: the charge repulsive force of the *j_th_*, *m_th_*_,_ and *n_th_* charged droplets to the *i_th_* charged droplet, respectively; *F_g_*: the gravity of the *i_th_* charged droplet.

During the electrospray process, the electrospray droplets in the high electric field are affected by the electric field force, the gravity, and the charge repulsive force, leading to the relative motion between the nozzle and the collecting plate. A dynamic model of electrospray droplets was constructed, as illustrated in Figure 1.

Some assumptions are presented in the work: (1) the charged droplet was fully atomized under the charge expansion pressure and hydrostatic pressure after being ejected from the Taylor cone tip; (2) each charged droplet was spherical, and the deformation in the process of droplet motion was ignored; (3) the charge and mass on the charged droplet were concentrated at the center of each spherical droplet; (4) the amount of charge carried by a charged droplet would not decay; (5) the influence of solvent volatilization on the droplet volume and interfacial layer properties was ignored; (6) the interference of environmental factors on the spatial motion of charged droplets as well as the effect of air resistance were ignored; (7) the influences of free charge transfer and strong electric field polarization on droplet motion were neglected.

#### 2.1.1. Electric Field Force

When the droplet was ejected from the tip of Taylor cone at the nozzle outlet, it took away most free charges on the surface of the solution. The electric field force on the *i_th_* droplet could be calculated as

(1) $${\vec{F}}_{Ei}=q\vec{E}$$

where *q* was the amount of charge carried by a single charged droplet, and $\vec{E}$ was the electric field strength at the space point where the droplet was located, which could be calculated according to the tip-plane model [19], as given by

(2) $$E=\frac{H\cdot C}{X\left(2H-X\right)+\left(H-X\right)a}$$

where *H* was the distance from the center of the nozzle tip to the collecting plate, *X* was the distance from the nozzle tip to the space electric field calculation point, *a* was the nozzle tip diameter, and *C* was the electric field constant, which was proportional to the applied voltage on the nozzle.

#### 2.1.2. Charge Repulsive Force

A large number of electrospray droplets was generated under the action of the high field between the nozzle and the collecting plate. The high-density homopolar free charge on the surface of the droplets led to a strong charge repulsive force among the droplets. The charge repulsive force of the *j_th_* (1 ≤ *j* ≤ *N* − *M*) charged droplet to the *i_th_* (1 ≤ *i* ≤ *N* − *M*) charged droplet in the space could be calculated as

(3) $${\vec{F}}_{qji}=-k\frac{q^{2}}{l_{j,i}^{2}}\vec{R}$$

where *l_j,i_* was the distance between the *i_th_* charged droplet and the *j_th_* charged droplet, *k* was the charge coulomb constant, $k=9.0\times 10^{9}\;N\cdot m^{2}{/C}^{2}$, and $\vec{R}$ was the vector identification, $\vec{R}=\frac{\left(x_{j}-x_{i}\right)\vec{x}+\left(y_{j}-y_{i}\right)\vec{y}+\left(z_{j}-z_{i}\right)\vec{z}}{\sqrt{{\left(x_{j}-x_{i}\right)}^{2}+{\left(y_{j}-y_{i}\right)}^{2}+{\left(z_{j}-z_{i}\right)}^{2}}}$.

Thus, the charge repulsive force from the spatial motion droplets on the *i_th_* charged droplet could be expressed as

(4) $${\vec{F}}_{qi1}=-k\overset{N-M}{\underset{j=1}{\sum }}\frac{q^{2}}{l_{j,i}^{2}}\vec{R}$$

where *M* was the number of droplets deposited on the collecting plate and *N* was the number of whole calculated number. 

Additionally, the droplets deposited on the collecting plate also have the same polarity charge as the droplets in the space, and there will be a charge repulsive force on the motion droplet as well [20]. The charge repulsive force between the *k_th_* deposited droplet and the *i_th_* motion droplet can be expressed as

(5) $${\vec{F}}_{qki}=-k\frac{q\cdot q_{kt}}{l_{k,i}^{2}}\vec{R}$$

where *l_k,i_* was the distance between the *k_th_* deposited droplet and the *i_th_* motion droplet, and *q_kt_* was the amount of charge of the droplets deposited on the collecting plate, which would be transferred to the ground with the deposition time, as calculated by

(6) $$q_{kt}=q\cdot e^{-\frac{t_{k}}{\tau }}/E_{c}$$

where *q* was the initial amount of charge on the droplet, *t_k_* was the time of the droplet reaching the collecting plate, *τ* was the relaxing time of the collecting plate, and *E_c_* was the electrical field strength inside the collecting plate.

Thus, the charge repulsive force from the whole deposited droplets on the *i_th_* charged droplet could be expressed as

(7) $${\vec{F}}_{qi2}=-k\overset{M}{\underset{k=1}{\sum }}\frac{q\cdot q_{kt}}{l_{k,i}^{2}}\vec{R}$$

Then, the total charge repulsive force on the *i_th_* charged droplet could be expressed as

(8) $${\vec{F}}_{qi}={\vec{F}}_{qi1}+{\vec{F}}_{qi2}$$

#### 2.1.3. Gravity

According to the law of conservation of mass, the mass of the charged droplet was constant during the electrospray process. Then, the gravity of the *i_th_* charged droplet could be expressed as

(9) $${\vec{F}}_{gi}=mg$$

where *m* was the mass of the charged droplet and *g* was the acceleration coefficient of gravity, $g=9.8{\;m/s}^{2}$.

### 2.2. Simulation Model of Droplet Motion

The fully resolved direct numerical simulation (FR-DNS) method [21] is a typical approach to describe the instant state of the spatial motion droplet, including the information of position, and the velocity by constructing the velocity evolution equation. Based on the force analysis, the dynamic equation of the electrospray droplets could be expressed as

(10) $$m\frac{d\vec{v_{i}}}{dt}={\vec{F}}_{Ei}+{\vec{F}}_{qi}+{\vec{F}}_{gi}$$

where *v_i_* was the motion velocity of the *i_th_* charged droplet.

**Figure 2:**
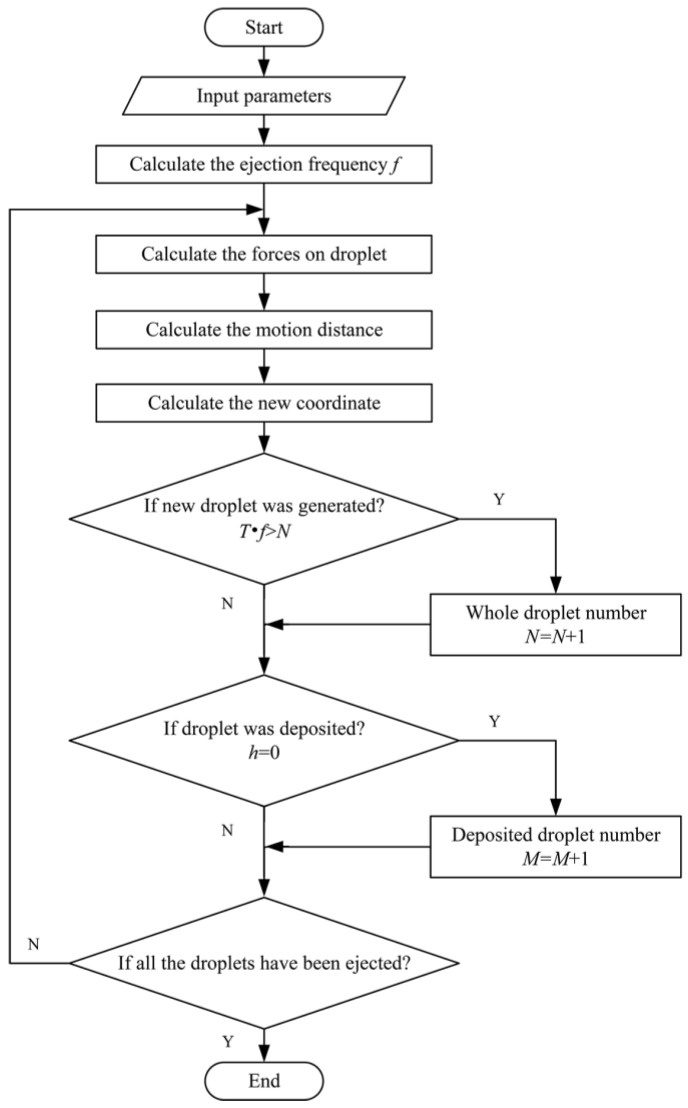
Flow chart of simulation program for droplet motion behaviors.

Then, a simulation program based on the Four Bands Runge–Kutta method was built to simulate the spatial distribution of electrospray droplets through the Matlab software (MATLAB R2022b, MathWorks, Natick, MA, USA). The flow chart of the simulation program is presented in Figure 2. 

The mass and charge density of droplets was determined by the break-up process of liquid, which was a competition between the liquid hydrostatic pressure, the surface tension, and the charge expansion pressure, synthesized by the liquid physical properties. A 2% polyethylene (PEO) solution with the solvent of deionized water and ethanol (*v/v* = 3:1) was selected as the electrospray material with viscosity and surface tension of 59.0 mPa·s and 40.8 mN/m. The electrospray droplets were captured via a high-speed camera (Miro M110, Phantom, NJ, USA) and the charged density was measured by a Coulomb meter (NK 1001, Kasuga, Kanagawa, Japan). Therefore, the charge on a single electrospray droplet could be estimated as the measured charge divided by the approximate droplet number within a unit of time. Thus, in our simulation model, the mass of a single droplet was set to be $m=10^{-12}\;\mathrm{kg}$, and the amount of charge carried by a single droplet was set to be $q=10^{-15}\;C$. The silicon substrate was used as the collecting plate, of which the relative dielectric coefficient and conductivity were set to be 11.9 and $4.35\times 10^{-6}$ S/cm. The generation period of droplets was set to be $T=10^{-4}\;s$. The simulation time step was set to be $10^{-8}$ s.

As the electric field strength was approximately $E\sim 10^{6}\;V/m$, and the initial distance between the nearest two droplets was approximately $l\sim 10^{-6}\;m$, the typical force scales on the electrospray droplets could be estimated as $F_{E}\sim 10^{-9\;}\;N$, $F_{q}\sim 10^{-9\;}\;N$, and $F_{g}\sim 10^{-11\;}\;N$. Thus, the electric field force and the charge repulsive force played the dominant cooperation effects on the motion and distribution behaviors of the electrospray droplets. The processing parameters including the applied voltage and the distance between the nozzle and the collecting plate were used as the inputs of the simulation program to analyze the effect of forces affecting the dynamic motion of droplets.

## 3. Results and Discussion

**Figure 3:**
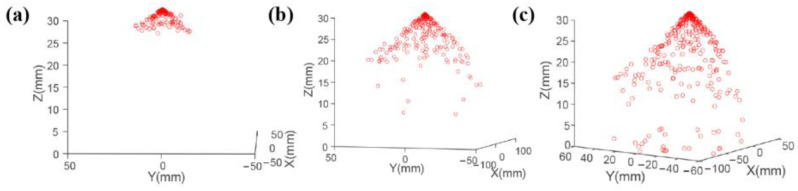
Simulation results of motion behavior for electrospray droplets: (**a**) 0.015 s; (**b**) 0.025 s; (**c**) 0.03 s. The applied voltage was 4 kV, and the distance from the nozzle to the collecting plate was 3 cm.

The motion behavior of the electrospray droplets was simulated, as shown in Figure 3. The droplets were ejected from the nozzle one by one according to the ejection frequency, then distributed in an “umbrella” shape in the space between the nozzle and the collecting plate. At the nozzle outlet, the motion velocity of the electrospray droplet was small, and the distribution density was high, while as the distance from the nozzle outlet increased, the motion velocity of the electrospray droplet increased, and the distance between the charged droplets increased, leading to the decrease in the distribution density and the increase in the distribution range of the electrospray droplets.

**Figure 4:**
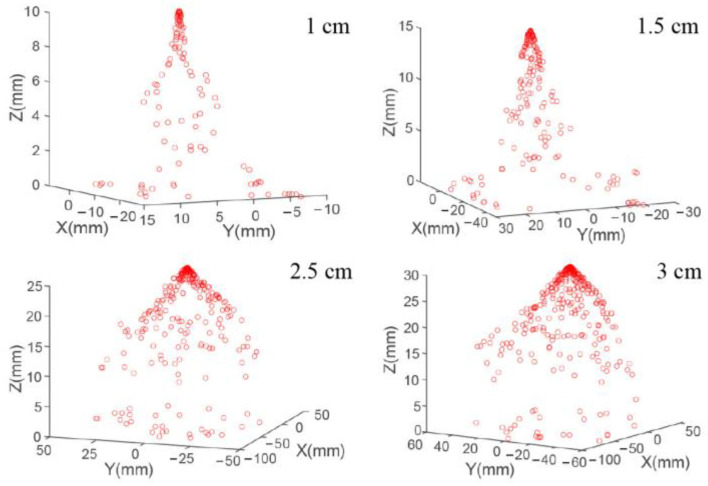
Simulation results of distribution for electrospray droplets under different distances from the nozzle to the collecting plate. The applied voltage was 4 kV.

The distributions of the electrospray droplets under different distances from the nozzle to the collecting plate and different applied voltages are shown in Figure 3 and Figure 4, respectively. The motion velocity of the electrospray droplet decreased with the increase in distance from the nozzle to the collecting plate due to a lower electrical field strength. As shown in Figure 4, when the distance from the nozzle to the collecting plate was small, the droplet moved under a large motion velocity to the collecting plate after being ejected from the nozzle, resulting in a small distribution density, while with the increase in distance from the nozzle to the collecting plate, the electrical field strength decreased, leading to a smaller motion velocity, thus the distribution density of the droplets at the nozzle outlet increased with the distance from the nozzle to the collecting plate. Additionally, the number of motion droplets also increased with the distance from the nozzle to the collecting plate, and the deposition area increased significantly.

**Figure 5:**
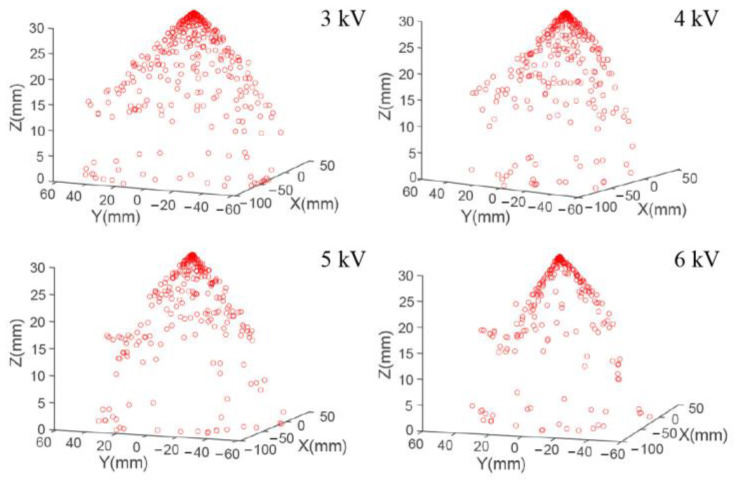
Simulation results of distribution for electrospray droplets under different applied voltages. The distance from the nozzle to the collecting plate was 3 cm.

As the electrical field strength increased proportionally with the applied voltage, the effect of electric field force on the droplet motion behavior was increased by accelerating the velocity of droplets. As shown in Figure 5, when the applied voltage was low, the motion velocity of the droplet ejected from the nozzle was small, leading to a high distribution density of droplets at the nozzle outlet and more motion droplets in the space. Thus, the distribution area of electrospray droplets increased as the charge repulsive force played the dominant effect. When the applied voltage was high, the droplets moved under a large motion velocity to the collecting plate after being ejected from the nozzle, and the influence of charge repulsive force was decreased as the electric field force acted with a dominant effect. In this way, the distribution density and deposition area decreased with the applied voltage.

**Figure 6:**
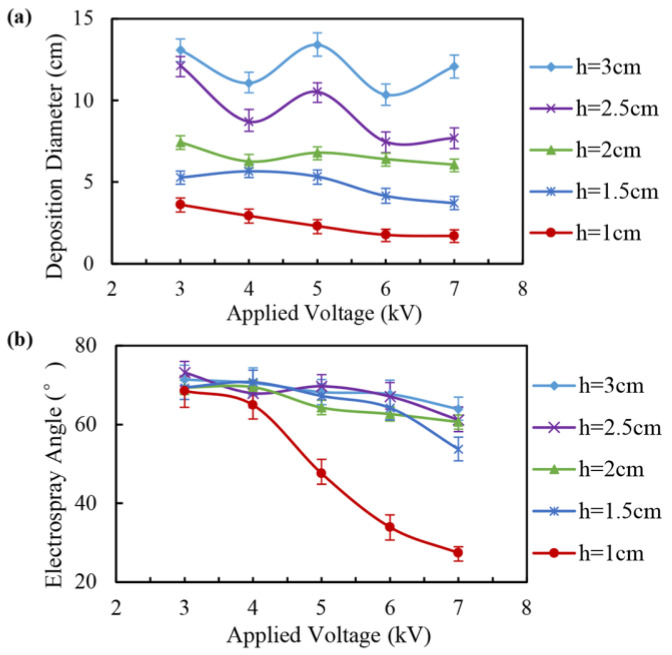
Distribution characteristics of charged droplets under different applied voltages and distances from the nozzle to the collecting plate (*h*): (**a**) deposition diameter; (**b**) electrospray angle.

The distribution characteristics of the electrospray droplets were also simulated, as shown in Figure 6. When the distance from the nozzle to the collecting plate was 1 cm, the deposition diameter decreased from 3.61 cm to 1.71 cm, and the electrospray angle decreased from 68.5° to 27.4° as the applied voltage increased from 3 kV to 7 kV. When the distance from the nozzle to the collecting plate was 1.5 cm, the deposition diameter decreased from 5.28 cm to 3.71 cm, and the electrospray angle decreased from 69.4° to 53.8° as the applied voltage increases from 3 kV to 7 kV. When the distance from the nozzle to the collecting plate increased further, the spatial electric field intensity decreased, and the motion velocity of the charged droplet decreased. Therefore, the influence of the charge repulsive force on the motion and distribution of charged droplets gradually became dominant. The deposition diameter increased significantly with the increase in the distance from the nozzle to the collecting plate. However, when the distance between the nozzle and the collecting plate was greater than 2 cm, the electrospray angle of the droplet changed little and was basically distributed between 60° to 75°.

**Figure 7:**
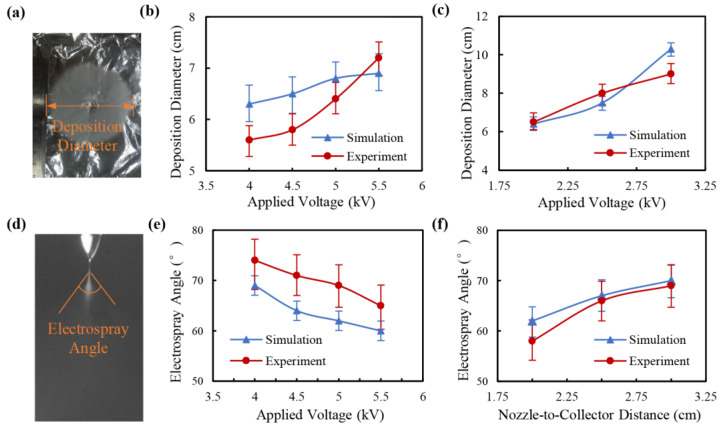
Experimental results of deposition diameter and electrospray angle compared with the simulation results. (**a**) Photograph of deposition membrane; (**b**) deposition diameter under different applied voltages. The distance from the nozzle to the collecting plate was 2 cm. (**c**) Deposition diameter under different distances from the nozzle to the collecting plate. The applied voltage was 6 kV. (**d**) Photograph of electrospray jet. (**e**) Electrospray angle under different applied voltages. The distance from the nozzle to the collecting plate was 2 cm. (**f**) Electrospray angle under different distances from the nozzle to the collecting plate. The applied voltage was 6 kV.

Electrospray experiments were conducted to verify the effectiveness of the simulation model, as presented in Figure 7. The deposition membrane is shown in Figure 7a and the electrospray jet is captured as Figure 7d. After being ejected from the nozzle, the electrospray droplets were distributed in an “umbrella” shape and deposited into a round membrane, as shown in the simulation results in Figure 4 and Figure 5. The deposition diameters and electrospray angles under different applied voltages and distances from the nozzle to the collecting plate were also investigated, as depicted in Figure 7b,c,e,f. The variation trends of the experimental results were like that of the simulation results, indicating that the numerical simulation method could guide the experimental process of electrospray. However, the electrospray droplets were modeled as charged rigid particles moving in the electric field, ignoring their fluidity (including the oscillation, the deformation, and the break-up process). Thus, the simulation approach could not fit the experiment results well.

The droplet interval is also an important characteristic to investigate regarding the distribution uniformity of electrospray droplets. Assume that the last droplet ejected from the nozzle was numbered as the 1st droplet, the penultimate droplet ejected from the nozzle was numbered as the 2nd droplet, …, and the first droplet ejected from the nozzle was numbered as the *N*_th_ droplet (*N* was the whole calculated droplet number), namely that the droplet number increased with the ejection distance. Then, the droplet interval could be defined as the distance between the *i*_th_ droplet and the (*I* + 1)*_th_* droplet:

(11) $$l_{i,i+1}=\sqrt{{\left(x_{i+1}-x_{i}\right)}^{2}+{\left(y_{i+1}-y_{i}\right)}^{2}+{\left(z_{i+1}-z_{i}\right)}^{2}}$$

where ($x_{i}$,$y_{i}$,$z_{i})$ and ($x_{i+1}$,$y_{i+1}$,$z_{i+1})$ were the coordinate of the *i_th_* droplet and the (*I* + 1)*_th_* droplet, respectively.

**Figure 8:**
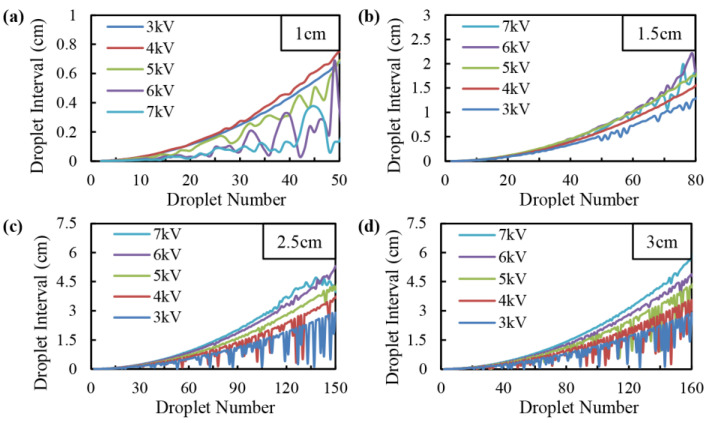
Effect of applied voltage on droplet interval under different distances from the nozzle to the collecting plate: (**a**) 1 cm; (**b**) 1.5 cm; (**c**) 2.5 cm; (**d**) 3 cm.

The effect of applied voltage on droplet interval under different distances from the nozzle to the collecting plate was simulated as Figure 8. It could be seen that the droplet interval increased with the ejection distance in a quadratic function relationship. When the distance from the nozzle to the collecting plate was 1 cm, the electric field force played the main role on the motion behavior of droplets rather than the charge repulsive force due to the short motion distance. Thus, the droplet interval decreased with the applied voltage, as depicted in Figure 8a. However, with the increase in the distance from the nozzle to the collecting plate, the motion velocity was decreased, and the effect of charge repulsive force was enhanced. As such, when the distance from the nozzle to the collecting plate was longer than 2 cm, the droplet interval increased with the applied voltage, as shown in Figure 8c,d.

The motion velocity of the droplet was directly determined by the electric field strength, which was increased by the applied voltage and decreased by the distance from the nozzle to the collecting plate. It should be noted that when the electric field strength was small, the effect of the charge repulsion force played a dominant effect and the motion velocity of droplets was small, thus the droplet interval would not increase in a direct way, especially at the stage far away from the nozzle. The larger the distance from the nozzle to the collecting plate, the higher the applied voltage needs to be to counteract the influence of the charge repulsion force on the motion of electrospray droplets.

From the simulation results, a quantitative relationship can be summarized between the droplet interval *l* and the ejection distance *d*:

(12) $$l\sim \beta d^{2}$$

where *β* was a coefficient related to the applied voltage and the distance from the nozzle to the collecting plate.

## 4. Conclusions

In this paper, a simulation method was built to investigate the motion and distribution behavior of electrospray droplets. The coupling effect of electric field force, the charge repulsive force, and the gravity was analyzed to construct the dynamic model. The motion and distribution of electrospray droplets under different applied voltages and distances from the nozzle to the collecting plate were simulated and presented in a direct way. The deposition diameter and the electrospray angle decreased with the applied voltage and increased with the distance from the nozzle to the collecting plate. The influencing factors of droplet intervals were also investigated. A quadratic function relationship was found between the droplet interval and the ejection distance. This work will promote the uniform and controllable deposition of electrospray droplets.

## Data Availability

The data presented in this study are available on request from the corresponding author. The data are not publicly available due to the research is still undergoing.
